# Temporal Control of Immediate Early Gene Induction by Light

**DOI:** 10.1371/journal.pone.0008185

**Published:** 2009-12-04

**Authors:** Philipp Schoenenberger, Daniela Gerosa, Thomas G. Oertner

**Affiliations:** Friedrich Miescher Institute for Biomedical Research, Basel, Switzerland; Vrije Universiteit Amsterdam, Netherlands

## Abstract

**Background:**

The light-gated cation channel channelrhodopsin-2 (ChR2) is a powerful tool for the optical induction of action potentials in neurons. Mutations of the cysteine 128 (C128) residue have been shown to greatly extend the lifetime of the conducting state of ChR2. However, until now, only subthreshold depolarizations have been reported from C128 mutants.

**Methods and Findings:**

Here we report the induction of long high-frequency spike trains by brief light pulses in ChR2(C128A)-transfected pyramidal cells in hippocampal slice culture. ChR2(C128A)-mediated spike bursts triggered expression of the immediate early gene c-fos in pyramidal neurons. Robust and cell-specific expression of c-Fos protein was detected after a single blue light pulse and depended on action potential firing, but not on synaptic activity. However, photocurrents diminished upon repeated stimulation and limited the number of action potential bursts that could be elicited.

**Conclusions:**

We conclude that the C128A mutant is not suitable for chronic stimulation of neurons, but very useful for light-controlled induction of immediate early genes. This property of ChR2(C128A) could be harnessed to control the expression of proteins under control of the c-fos promoter with precise timing and single cell specificity.

## Introduction

Optogenetic control of neuronal firing has become a widely used tool to manipulate the activity of single neurons or neuronal ensembles *in vitro* and *in vivo* [1]–[4]. Since the advent of the light-gated cation channel channelrhodopsin-2 (ChR2) in the neurosciences [5], the optogenetic toolbox has been steadily extended. For example, ChR2 mutants with greatly extended lifetimes of the open channel state have been generated. These ‘bi-stable’ channelrhodopsins are based on point mutations at the C128 position and elicit long-lasting photocurrents that can be switched off by a green light pulse [6]. Three different point mutations at the C128 position have been shown to give rise to ChR2 variants with slow channel closing kinetics: ChR2(C128T) has a closing time constant of 2 s, whereas ChR2(C128A) and ChR2(C128S) are characterized by even slower channel closure and time constants of 52 s and 106 s, respectively [6]. The C128A mutant has very interesting kinetics in that its open state outlast the activation light pulse by many orders of magnitude but it still spontaneously inactivates within an experimentally accessible time window. In their original publication, Berndt and colleagues used the C128A mutant to induce long and reversible subthreshold depolarizations in dissociated neurons and to sensitize cells to synaptic input [6].

In this study, we characterized the effects of ChR2(C128A) activation when the mutant channel is expressed at high levels in pyramidal cells in hippocampal slice culture. Photocurrents were very large initially, leading to high frequency spike trains. However, due to incomplete recovery of photocurrents in the dark, the number of spike trains that could be induced successively was limited. In the second part of the study, we focused on light-triggered expression of c-fos, an immediate early gene that has been used to map neural activity for more than two decades [7], [8]. Genetic activity reporters based on c-fos have allowed to follow the fate of cells activated during learning [9] and to investigate the trafficking of newly synthesized AMPA receptors [10]. We show that ChR2(C128A)-triggered spike trains reliably induce c-Fos expression in pyramidal neurons. We propose that co-expression of ChR2 mutants with activity reporters could be used to study activity-related processes *in vitro* and *in vivo*.

## Results

### Stability of ChR2(C128A) Photocurrents in Hippocampal Pyramidal Neurons

**Figure 1:**
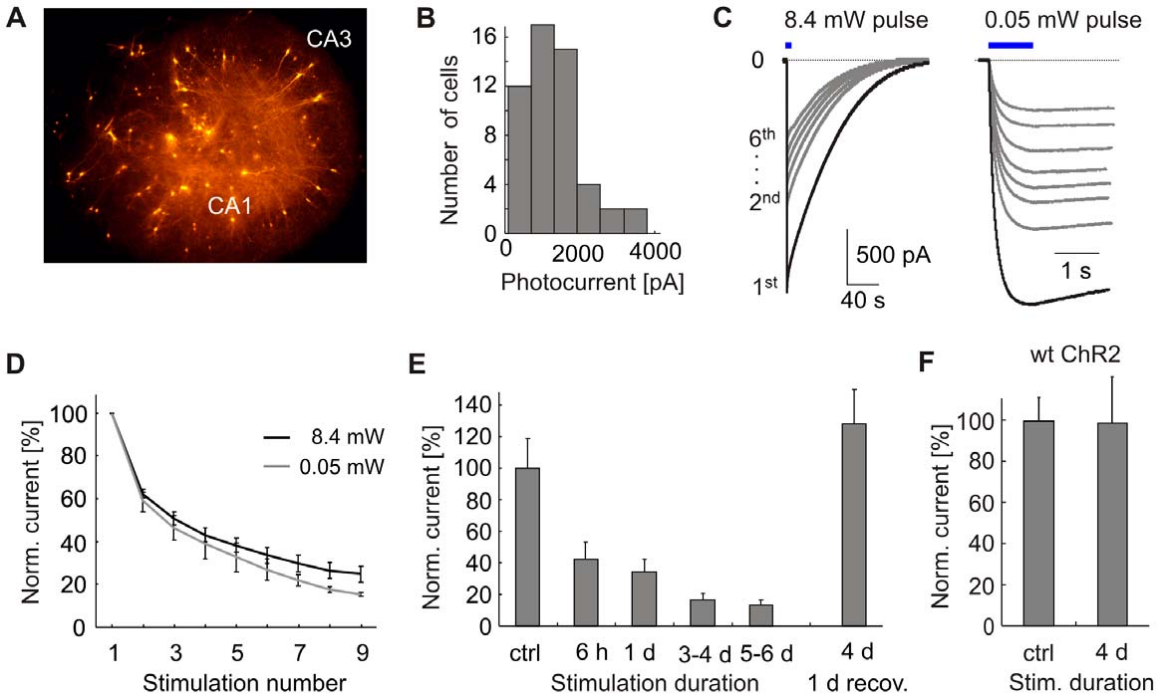
ChR2(C128A) photocurrents in hippocampal pyramidal neurons. (A) Rat organotypic slice culture co-transfected with ChR2(C128A) and cytoplasmic RFP. (B) Light-induced currents in voltage-clamped pyramidal cells (n = 52). Cells were stimulated using blue 50 ms light pulses (8.4 mW), current measurements were performed in the presence of 1 µM TTX, 10 µM NBQX, and 10 µM bicuculline. (C) Repeated stimulation of pyramidal neurons with high (8.4 mW for 50 ms; left) or low (0.05 mW for 1000 ms; right) light intensity. Blue bars indicate light stimulation pulses. (D) Photocurrent reduction with repeated stimulation at high or low light intensity (n = 6, 3, resp.). (E) Population comparison of photocurrents in non-stimulated cells (ctrl, n = 10) or cells stimulated in the incubator prior to photocurrent measurement (50 ms blue LED pulses at 90 s intervals, n = 6, 5, 18, 10). Photocurrents in cells stimulated for 4 d fully recovered within 24 h in the dark (n = 9). (F) No photocurrent reduction was observed in 4 d stimulated cells expressing wt ChR2 (n = 8) compared to non-stimulated control cells (n = 11).

We used the human synapsin 1 promoter to drive expression of ChR2(C128A) specifically in neurons. Using particle-mediated gene transfer, individual neurons in rat organotypic hippocampal slice cultures were co-transfected with ChR2(C128A) and the red fluorescent protein tdimer2 as a cytosolic marker (Fig. 1A). To asses ChR2(C128A)-mediated currents, we recorded from pyramidal cells stimulated with 50 ms blue light pulses from a mercury arc lamp. Photocurrents were isolated by bath application of NBQX and bicuculline to block synaptic input and TTX to block fast Na^+^ channels. The average photocurrent in cells voltage-clamped at −65 mV was 1325±110 pA (*n* = 52; Fig. 1B), considerably larger than reported previously [6]. When a cell was repeatedly stimulated, photocurrents were strongly reduced (Fig. 1C), although inter-stimulus intervals (ISIs) were sufficiently long to allow for full inactivation of the photocurrent (2.5–4.0 min). This effect was comparable for high (8.4 mW, 50 ms) and low (0.05 mW, 1000 ms) light intensities despite a reduction of the total light dose by a factor of 8.4. The response to identical light pulses was reduced after each stimulation pulse, dropping to 24.7±3.7% of the initial amplitude after 9 repetitions for bright pulses (*n* = 6; Fig. 1D) and 15.9±0.8% for low intensity pulses (*n* = 3). Further increasing the light dose by using longer light pulses (200 ms, 8.4 mW) did not lead to a stronger reduction of peak currents (75.3±4.0% reduction after 8 stimulations (*n* = 5), compared to 73.7±3.6% with 50 ms pulses), suggesting that photocurrent reduction was not a light dose-dependent phenomenon such as the bleaching of fluorophores.

Could the photocurrent reduction be related to cytosolic wash-out during whole-cell recordings? To test this possibility, we stimulated transfected cultures inside the cell culture incubator using blue high-power LEDs (50 ms pulses, 90 s intervals) prior to photocurrent measurements. Light-induced currents were reduced after only a few hours of stimulation, and the reduction was even more pronounced after several days (Fig. 1E). This indicates that the photocurrent reduction we observed was not caused by whole-cell dialysis. A dark period of 24 h prior to electrophysiological recording was sufficient for full recovery of photocurrents, showing that the decrease in current amplitude was not permanent but slowly reverted in the dark within several hours (Fig. 1E). Photocurrents in cells expressing wild type ChR2 were not reduced after 4 d stimulation (Fig. 1F), again indicating that ChR2(C128A) photocurrent run-down was not caused by photodamage to the chromophore but due to the extremely slow kinetics of the mutant channel. Together, these data show that ChR2(C128A) can generate very large photocurrents, and that repeated activation of the channel rapidly reduces the amplitude of light-triggered currents.

### Loss of Functional Channels Occurs during Relaxation from the Open State

**Figure 2:**
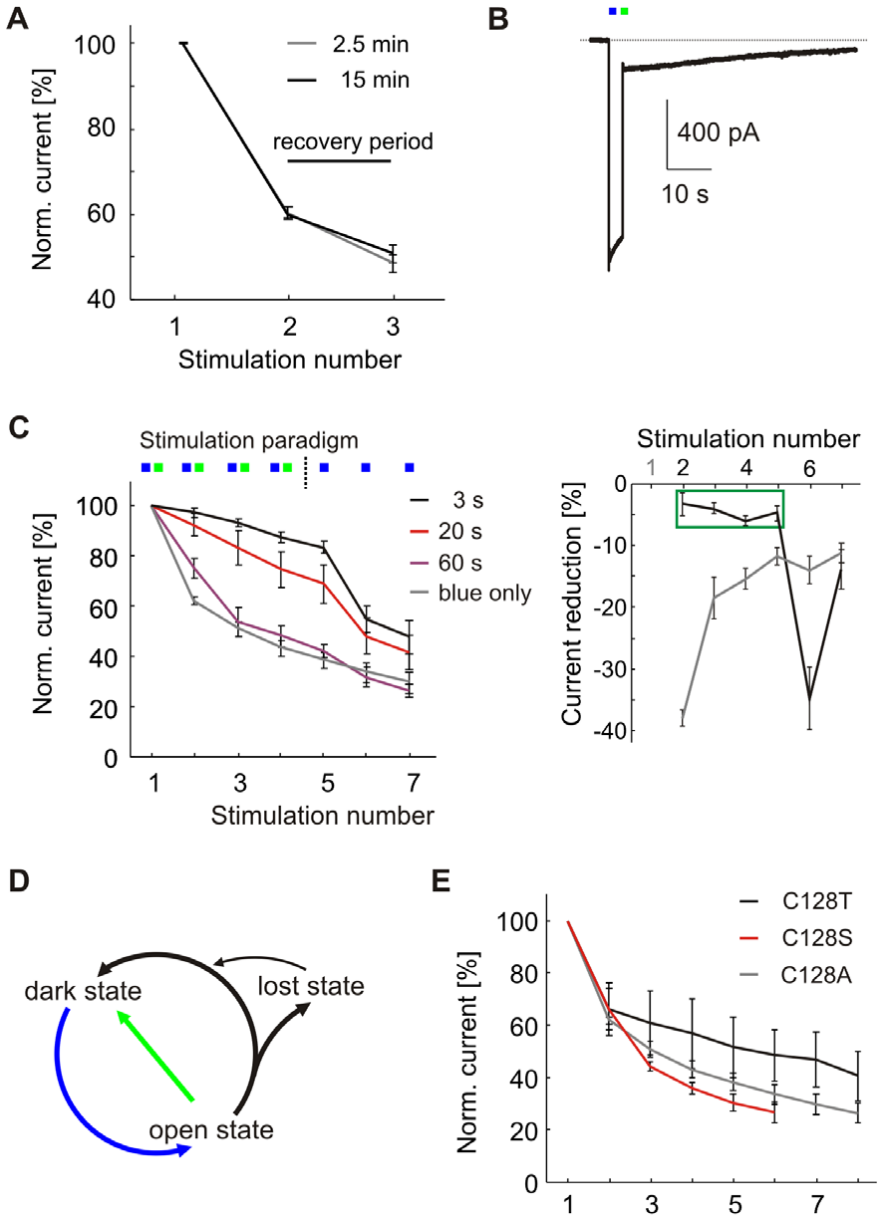
Photocurrent reduction can be alleviated by green switch-back pulses. (A) Neurons were stimulated with two blue pulses spaced 2.5 min apart. After a 2.5 min (gray, n = 10) or 15 min (black, n = 4) recovery period photocurrent reduction was comparable (p>0.5). (B) Following excitation by a blue light pulse, ChR2(C128A) photocurrents could be inactivated by a green switch-back pulse. Inactivation efficiency was 90.6±1.2% (n = 24). (C) Left: Photocurrent reduction was markedly diminished when activated channel was inactivated by a green switch-back pulse after a 3 s interval (black line). Application of a green light pulse after 20 s or 60 s was less effective in preventing current reduction (n = 3, 3). Gray line: Current reduction with single blue pulses for comparison. Right: Quantification of photocurrent reduction relative to the previous stimulation pulse without green switch-back (gray line) or with a green light pulse applied 3 s after blue light stimulation (black line). The green box indicates stimulation trials following a trial terminated with a green pulse. (D) Minimal model for the ChR2(C128A) photocycle, modified after Berndt et al. [6]. (E) Photocurrent reduction with repeated stimulation was also observed in ChR2(C128T) and ChR2(C128S) mutants.

Work on the ChR2 photocycle suggested that activated channels relax to the dark state via one or two relatively long-lived closed intermediate states [11], [12]. We wanted to determine whether the run-down of photocurrents was caused by a fraction of activated channels entering a non-functional state after each stimulation or by very slow recovery of the dark state after channel closure. In the latter case, we would expect a recovery of photocurrent amplitude that is proportional to the interval between subsequent pulses. To address this issue, we stimulated neurons with three blue light pulses. The first two pulses were spaced 2.5 min apart, sufficient for inactivation of photocurrents between pulses. The 3^rd^ light pulse was applied after a dark period of 2.5 min (*n* = 10) or 15 min (*n* = 4). The reduction in peak photocurrent in response to the 3^rd^ light pulse was independent of the length of the preceding dark period (current after 3^rd^ pulse: 48.5±2.1%, 50.7±2.0%, resp., p>0.5; Fig. 2A), suggesting that a fraction of channels was available for activation directly after channel closure whereas the remaining fraction entered a long-lived closed state that did not recover to the dark state within 15 min. For simplicity, we refer to this non-functional state as ‘lost’ state (Fig. 2D).

Could the loss of functional channels be alleviated by rapidly switching open channels back to the dark state using a green light pulse [6]? We stimulated pyramidal cells with a blue light pulse followed by a green pulse after 3 s, which reduced the photocurrents to 9.4±1.2% (*n* = 24; Fig. 2B). Residual current was allowed to fully inactivate before the next stimulation (ISI>2.5 min). This switch-back procedure markedly reduced the run-down of photocurrents upon repeated stimulation (16.7±2.3% reduction after 5 pulses, *n* = 6, compared to 66.5±3.8% without green switch-back; Fig. 2C). As expected, a single blue pulse applied after a sequence of blue - green switches led to strong reduction of peak photocurrents at the next pulse (Fig. 2C). Application of the green light pulse 20 s or 60 s after the blue stimulation pulse was less effective in preventing photocurrent reduction. We conclude that loss of functional ChR2(C128A) channels occurred during relaxation from the open state - either directly from the open state or from a closed intermediate - and could be strongly reduced by rapid optical channel closure (Fig. 2D).

To determine whether photocurrent reduction after repeated stimulation is a property that is specific for ChR2(C128A) we also generated and tested the other two bi-stable ChR2 mutants reported by Berndt and colleagues [6]. We extended the ISI to 6 min to account for the very slow inactivation of ChR2(C128S) and we maintained an ISI of 2.5 min for ChR2(C128T). Indeed, rapid and pronounced photocurrent loss upon repeated stimulation was also observed for the C128T and the C128S mutants indicating that photocurrent run-down could be a general feature of ChR2 variants (Fig. 2E).

### Properties of Light-Triggered Spike Trains in Pyramidal Neurons

**Figure 3:**
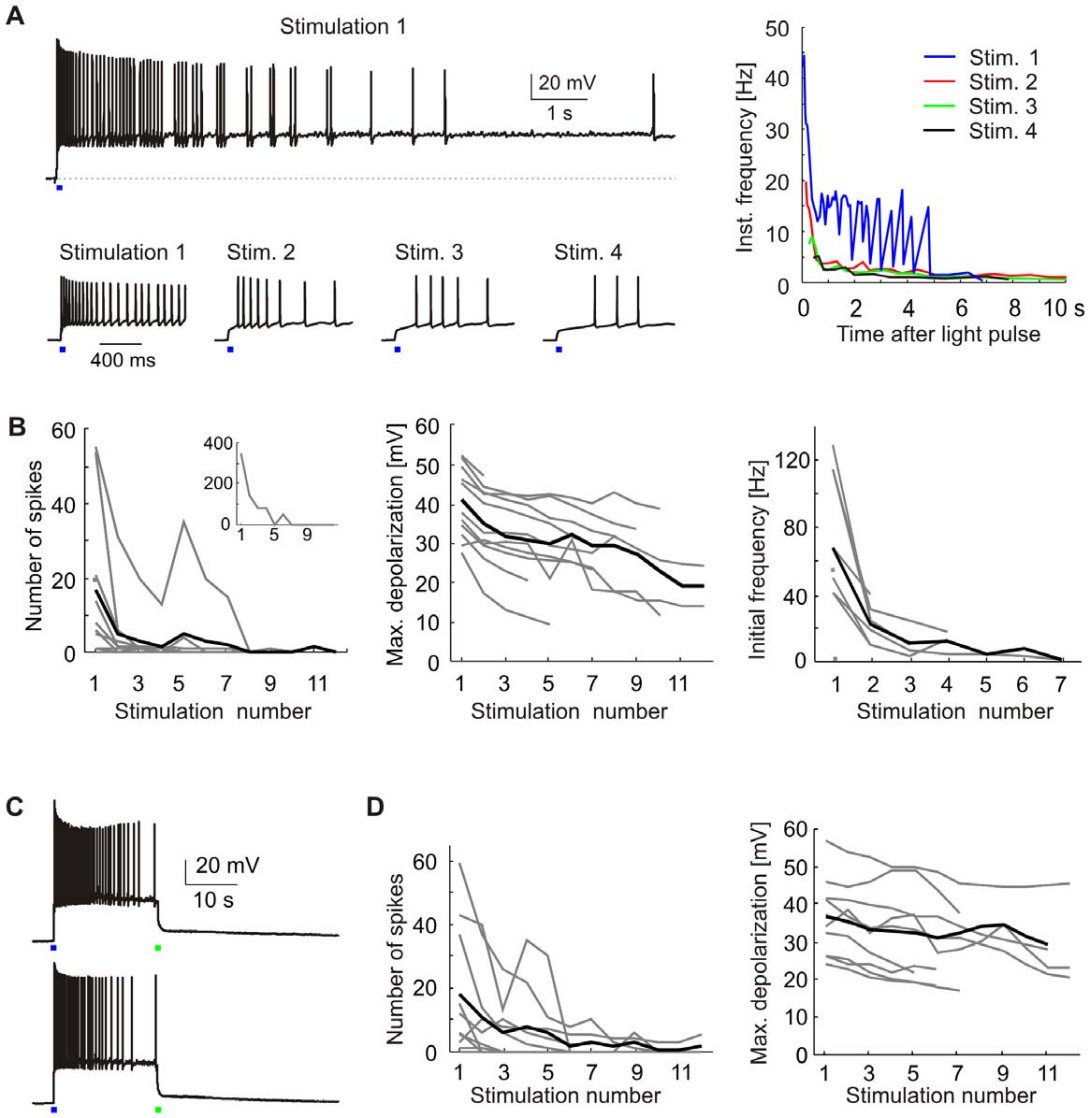
Light-induced spike trains in pyramidal neurons. (A) Spike trains triggered by four subsequent blue stimulation pulses. Depolarization was allowed to revert to baseline between stimulations. Right: Instantaneous frequency of spike trains. (B) Analysis of light-triggered spike trains. The number of spikes, maximal depolarization, and initial frequency decreased with repeated stimulation. Insert in first panel shows cell firing 355 APs after first stimulation pulse. Gray lines represent 12 individual cells, average is shown in black. (C) Light-induced depolarizations were interrupted by a green switch-back pulse after 20 s. Traces depict two subsequent spike trains. (D) The reduction in spike numbers and maximal depolarizations was comparable to experiments without green switch-back pulse (n = 10).

Next, we investigated ChR2(C128A)-triggered spike trains in hippocampal pyramidal cells in current-clamp. To reduce variability between trials, we adjusted the resting potential to −60 mV by injection of a small holding current. To prevent spontaneous network activity, we blocked excitatory transmission by NBQX. In response to a brief blue light pulse (50 ms), transfected cells fired a burst of action potentials (APs) (Fig. 3A). Subsequent light pulses evoked a steadily decreasing number of APs, indicating a reduction of the underlying photocurrents (Fig. 3B). We also observed a reduction in depolarization between spikes and a decrease in the initial firing frequency with repeated stimulation, consistent with a run-down of photocurrents. Next, we tested whether the number of spike trains fired by a cell could be increased by interrupting the light-triggered depolarization using a green light pulse applied 20 s after each blue pulse (Fig. 3C). We found that both the number of stimulations that triggered spikes and the reduction of the maximal depolarization were comparable to stimulation with blue light only (Fig. 3D), suggesting that the reduction in photocurrent loss was too weak to have a pronounced effect on spike train firing. Thus, it appears that the run-down of photocurrents upon repeated stimulation limited the number of light-triggered spike trains in ChR2(C128A)-expressing cells.

**Figure 4:**
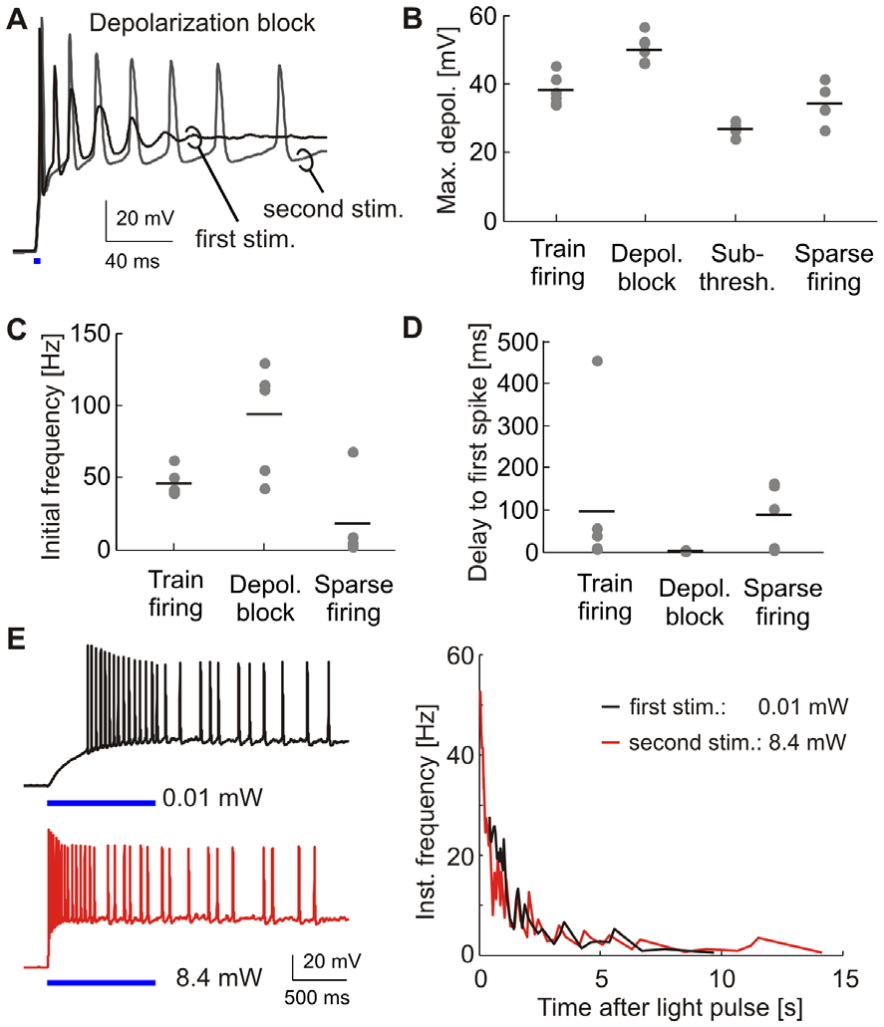
Analysis of light-triggered activity in current-clamped cells. (A) Sample traces of a cell entering depolarization block after the first stimulation pulse. Note the pronounced spike amplitude attenuation. The second pulse induced a smaller depolarization and a series of APs. (B – D) Spike train parameters. Cells were classified based on their response to the first stimulation pulse. Train firing: >30 APs, n = 6. Depolarization block, n = 7. Subthreshold depolarization, n = 4. Sparse firing: 1–30 APs, n = 5. (E) Spike trains in a pyramidal neuron stimulated with very low or high light intensity. Analysis of the instantaneous firing frequency reveals a similar rapid frequency drop under both conditions.

Why did some pyramidal cells fire long spike trains whereas others fired only single spikes or failed to fire APs altogether? We classified cells based on their response to the first stimulation pulse into four categories (Fig. 4): Cells that did not reach firing threshold (*n* = 4), sparse firing (1–30 APs; *n* = 5), train firing (>30 APs, *n* = 6), and cells entering depolarization block (*n* = 7). Cells entering depolarization block were defined by strong spike amplitude attenuation due to incomplete repolarization between spikes (Fig. 4A). The range of depolarizations leading to train firing was very narrow (38.2±1.8 mV; Fig. 4B), as was the range of initial firing frequencies in this group (45.8±3.5 Hz; Fig. 4C). Cells entering depolarization block were characterized by strong depolarization (Fig. 4B), high initial firing frequencies - in some cases exceeding 100 Hz (Fig. 4C) - and very short delays to first spike (4.2±0.4 ms; Fig. 4D). In summary, responses of individual pyramidal cells were quite variable; consistent with the large variability of photocurrents we measured (Fig. 1B).

An attractive property of the slow ChR2 mutants is their enhanced light sensitivity, which could enable light stimulation *in vivo* without the need to implant fiber optics. We were interested whether extremely low light intensities would induce different firing patterns in ChR2(C128A)-expressing neurons. We first stimulated cells with a long but very dim light pulse (0.01 mW, 1000 ms). A second stimulation pulse with high light intensity (8.4 mW, 1000 ms) was applied after a 2.5 min interval (Fig. 4E). To minimize current loss between stimulation trials, light-induced depolarization was terminated by applying a green pulse 20 s after stimulation. Bursts of APs were fired in response to stimulation with either low or high light intensity in 5 cells we recorded from. Interestingly, the number of spikes was very similar under both stimulation conditions (0.01 mW: 22.8±5.1 APs; 8.4 mW: 23.6±7.2 APs). However, due to the slow depolarization with low light intensity, the first spike was fired after a delay of 393±64 ms whereas the delay was only 43±16 ms for high light intensity. The firing pattern of a given cell was similar after low and high intensity light stimulation. The initial firing frequency was higher with a bright stimulation pulse and thus rapid depolarization, but the firing frequency rapidly dropped independently of the stimulation condition (see Fig. 4E, right). When the stimulation paradigm was repeated the number of APs per stimulation trial rapidly decreased (after 6 repetitions: 0.01 mW: 1.4±1.3 APs; 8.4 mW: 2.8±1.9 APs). These results indicate that ChR2(C128A) can reliably induce AP firing even at very low light intensities, a property that may be useful to activate cells deep within the brain.

### Recording of Light-Triggered Responses in Cell-Attached Mode

**Figure 5:**
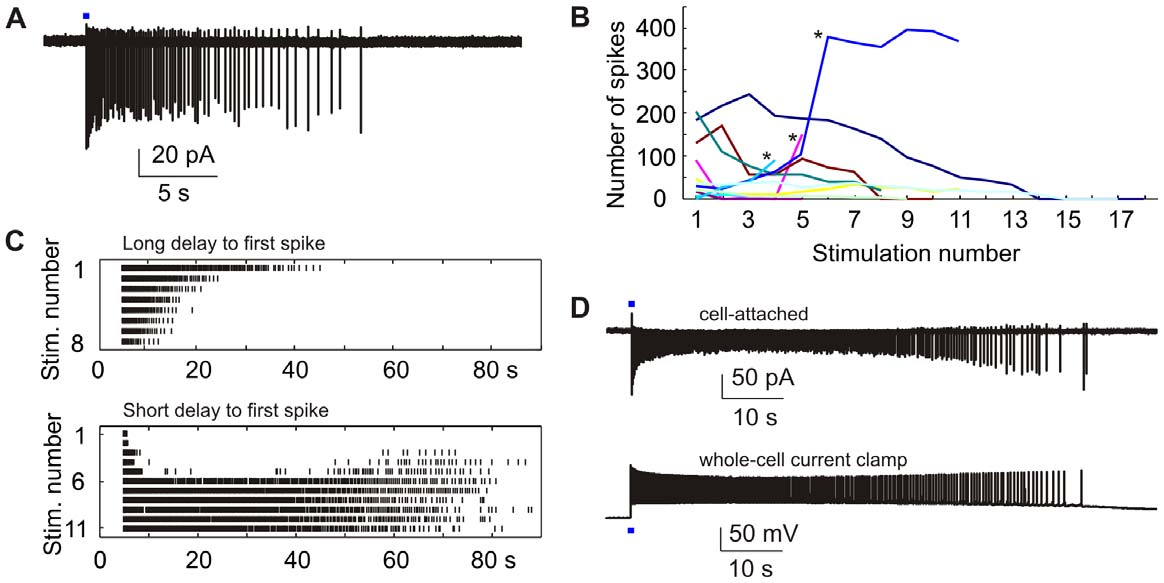
Cell-attached recordings of light-induced spike trains. (A) Sample trace. Cell-autonomous activity was isolated by bath-application of NBQX. (B) In the majority of cells the number of spikes decreased with repeated stimulation (8/11 cells). Asterisks indicate cells presumably entering depolarization block after initial stimulation pulses. (C) Top: Spike raster for cell with long delay to first spike (10.75 ms). Bottom: Spike raster for cell with short delay to first spike (4.25 ms) presumably entering depolarization block. (D) Top: Long spike train containing 394 APs. Bottom: Spike train in current-clamped cell (355 APs) for comparison.

To characterize light-triggered activity in unperturbed pyramidal neurons, we performed a series of cell-attached recordings (Fig. 5A). Similar to whole cell recordings, the number of APs decreased with repeated stimulation in most cells (8/11 cells; Fig. 5B and C (top)). We also observed cells that fired an increasing number of spikes upon repeated stimulation (3/11 cells; Fig. 5B and C (bottom)). During the first stimulations, these cells fired a brief high frequency burst with pronounced spike amplitude attenuation immediately after light onset (average delay: 4.0±0.7 ms), and a few more spikes after a silent period of 20–60 s (Fig. 5C, bottom). In these cells, run-down of photocurrents paradoxically led to an increase in total spike output in subsequent stimulations, suggesting that during the first stimulations, they entered depolarization block. Qualitatively, our cell-attached recordings thus confirmed the results of light stimulation in whole-cell configuration: Similar response classes were found, and long bursts of APs with decreasing frequency were observed in both recording configurations (Fig. 5D). Quantitatively, spike trains were often longer in cell-attached recordings, indicating that whole-cell recording slightly impeded AP generation.

### Cell-Autonomous c-Fos Induction by Light-Triggered Action Potential Firing

**Figure 6:**
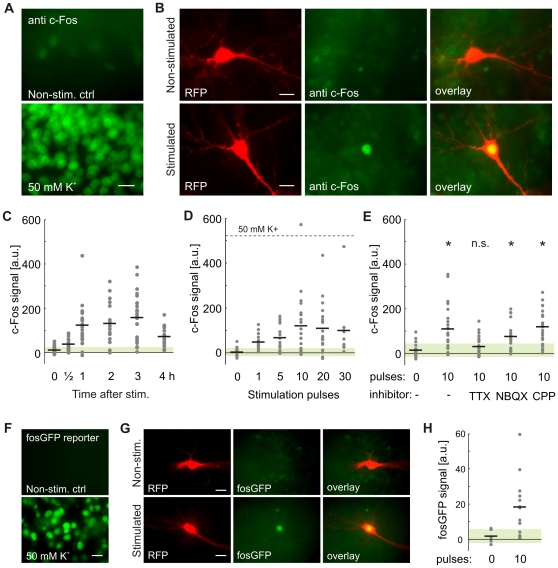
Cell-specific c-Fos induction by light-triggered spike trains. (A) Immunostaining for endogenous c-Fos in CA1 area of rat hippocampal slice cultures under basal conditions (top) and 2 h after stimulation by extracellular application of 50 mM K^+^ (bottom). (B) c-Fos induction in a pyramidal neuron expressing ChR2(C128A) and cytosolic RFP after 10 stimulation pulses. Cultures were fixed 2 h after stimulation and stained for c-Fos. (C) c-Fos signal in non-stimulated cells (n = 27) and at different time points after light stimulation (n = 18, 24, 18, 25, 21). Green shaded bar indicates mean±SD of non-stimulated control cells. c-Fos induction was significant at all time points (p<0.01). (D) Significant c-Fos induction was observed after a single blue light pulse (n = 17, 15, 14, 21, 19, 11). Dashed line indicates average c-Fos signal after 50 mM K^+^ stimulation. (E) Light-triggered c-Fos induction was blocked by TTX, but not by NBQX or CPP. *: p<0.01 compared to control. (F) Green fluorescence in live organotypic slice cultures (CA3) from fosGFP transgenic mice under basal conditions (top) and 4 h after brief stimulation with 50 mM K^+^ (bottom). (G) Light-induced GFP expression in fosGFP reporter mouse neurons expressing ChR2(C128A) and RFP. (H) Light-induced GFP expression was quantified 4 h after light stimulation (10 pulses). Scale bars in (A,B,F,G): 10 µm.

To investigate the cellular consequences of ChR2(C128A)-induced spike bursts, we stained stimulated and non-stimulated rat hippocampal slice cultures for endogenous c-Fos protein. Under basal conditions, c-Fos expression was very low (Fig. 6A). Exposing the cultures to 50 mM K^+^ (3×2 min, 10 min intervals) led to strong c-Fos induction 2 h after stimulation (Fig. 6A). To test for light-induced expression of c-Fos, we stimulated transfected cultures in the cell culture incubator with LED-generated light pulses (300 ms pulse length, ISI = 90 s). In a first set of experiments, we fixed cultures at different time points after stimulation with 10 light pulses and stained for c-Fos (Fig. 6B). Interestingly, c-Fos levels were already significantly increased after 30 min (p<0.01; Fig. 6C). After 1 h, c-Fos expression reached a plateau that was maintained for about 2 h.

Next, we stimulated cultures with different numbers of stimulation pulses. We found that a single blue light pulse was sufficient to induce c-Fos expression in transfected pyramidal cells (p<0.03 compared to non-stimulated control; Fig. 6D). After a single light pulse, c-Fos levels were heterogeneous: Significant c-Fos upregulation (signal larger than mean + 2 SD of control) was detected in 7/15 cells. After 10 light pulses, maximal optical c-Fos induction was reached. High K^+^ stimulation induced even stronger c-Fos signals (Fig. 6D, dotted line). We conclude that the lack of further c-Fos upregulation by additional light pulses was most likely due to run-down of photocurrents, limiting the number of spike trains triggered in individual neurons. Importantly, induction of c-Fos after light stimulation was restricted to transfected cells (see Fig. 6B).

Light-triggered depolarizations were typically very large (Fig. 4B), raising the possibility that action potentials were not required to induce immediate early gene expression. We tested the possibility that calcium influx through ChR2 itself and through low-voltage-activated calcium channels was sufficient for c-Fos induction. Application of the Na^+^ channel blocker TTX during stimulation blocked c-Fos induction, indicating that spiking activity was necessary to trigger the signaling cascade leading to c-Fos expression (Fig. 6E). On the other hand, glutamate receptor antagonists (NBQX, CPP) did not impair light-induced c-Fos expression, indicating that synaptic activity was not required to induce c-Fos. These results validate the use of c-Fos expression as an indicator for cellular activity [8], [13]. Moreover, these data show that calcium influx through the ChR2 pore itself (see [3]) is not sufficient to induce c-Fos expression.

In order to investigate if ChR2(C128A) could be used to control c-fos promoter-driven transgene expression, we prepared hippocampal slice cultures from fosGFP transgenic mice [21]. These mice express a fosGFP fusion protein and have been used to identify and characterize recently activated neurons in live brain tissue [21]. Under basal conditions, virtually no fosGFP signal was detectable. Brief stimulation with 50 mM K^+^ (3×2 min, 10 min intervals) induced strong fosGFP expression in pyramidal neurons (Fig. 6F). Light stimulation of cultures transfected with ChR2(C128A) and RFP (300 ms pulse length, 10 pulses, ISI = 90 s) led to strong and significant expression of the fosGFP reporter protein specifically in transfected neurons (Fig. 6G,H; p<0.05). As a control, we verified that transfected but not light-stimulated cells did not express GFP above background. Taken together, our data show that light-triggered spike bursts reliably induced c-Fos expression in a cell-specific and cell-autonomous manner. Moreover, light-induced spike trains can be exploited to control the expression of custom transgenes driven by the c-fos promoter.

## Discussion

Here we report that hippocampal pyramidal neurons expressing the bi-stable channelrhodopsin mutant ChR2(C128A) fire long spike trains in response to brief flashes of blue light. During repeated activation, pronounced reduction of photocurrent amplitudes limited the number of spike trains that could be triggered in individual cells. Downstream of spiking activity, we found that a single blue light pulse was sufficient to induce expression of the immediate early gene c-fos in about half of the transfected cells. We propose that the specific properties of ChR2(C128A) could be exploited for temporally controlled cell-specific induction of transgenes under the control of the c-fos promoter.

An unexpected property of ChR2(C128A) was the rapid decrease of peak photocurrent amplitude and bursting activity during repeated light stimulations. In fact, our first set of photostimulation experiments were not successful (data not shown), because a diffuse white LED we used to position the slice culture under the microscope already induced permanent inactivation of most of the photocurrent. This cautionary tale provides a potential explanation why a previous study of the very light-sensitive ChR2(C128A) reported only subshreshold depolarization and illustrates the practical importance of our findings [6]. In this context, it is important to note that we used RFP instead of YFP as a fluorescent marker and thus avoided blue light exposure while searching for transfected cells, thereby preventing loss of photocurrent.

The loss of photocurrent with repeated stimulations was not observed in cells expressing wt ChR2. Moreover, photocurrent run-down did not depend on the blue light dose applied in each stimulation trial. Therefore, we can exclude bleaching processes such as destruction of the chromophore (*all-trans* retinal) as a cause for run-down of light-induced currents. For inter-stimulus intervals up to 15 min, there was no recovery of photocurrents. Loss of photocurrent could be largely prevented by rapidly closing the channels with green light, confirming that the light-gated channel was not degraded by the blue light used for stimulation (Fig. 2C). We conclude that the time spent in the open state is the critical parameter that determines the fraction of channels lost in every trial. For wt ChR2, relatively long-lived closed channel intermediates have been described [11], [12], raising the possibility that we stimulated the cells at too short ISIs, preventing full recovery of the dark state between trials. However, prolonging the ISI from 2.5 to 15 min resulted in very similar reduction of peak photocurrent, arguing against a single rate-limiting step in a linear photocycle (Fig. 2A). More likely, the photocycle of ChR2(C128A) branches during relaxation from the open state, causing accumulation of more and more channels in a non-functional ‘lost’ state (Fig. 2D). Indeed, after 24 h in the dark, photocurrents did recover, indicating that spontaneous transitions from the lost to the dark state were very rare. Branched photocycles are not a novel concept, but have been proposed for various rhodopsins [14]–[16]. Future spectroscopic studies of the ChR2(C128A) photocycle may help to understand the nature of the lost state and to develop strategies to minimize photocurrent reduction upon repeated stimulation. Photocurrent run-down was not restricted to ChR2(C128A), but was also observed after repeated stimulation of ChR2(C128T) and ChR2(C128S) mutants, indicating that this phenomenon might be a general feature of ChR2 variants (Fig. 2E).

Our findings suggest that ChR2(C128A) is best suited for the temporally controlled induction of a limited number of strong activity bursts. The potential for chronic stimulation is limited at present, but might improve in the future as more information becomes available about the channel photocycle. A very interesting application would be light-triggered protein expression in selected cells. Genetic activity reporters based on the activity-dependent activation of the c-fos promoter have been used for in vivo activity mapping [9], [13] or for activity-induced transgene expression [10]. Combining selective ChR2(C128A) expression using suitable promoters with the spatial precision of light activation [17] will allow to use light pulses for precisely timed induction of c-fos promoter-controlled transgenes in specific cells. In contrast to methods for conditional gene expression relying on caged molecules [18]–[20], no addition of chemicals would be needed for optogenetic induction, an important advantage for *in vivo* applications. Indeed, we observed reliable light-triggered induction of a c-fos promoter-driven reporter protein in ChR2(C128A)-transfected neurons in cultures from transgenic mice, demonstrating the successful implementation of this approach (Fig. 6G,H). In summary, ChR2(C128A) seems ideally suited to trigger gene expression in single cells with high temporal precision.

Although c-Fos has been used in many studies as an activity reporter [8], [13], [21], [22], it is unclear what the minimal trigger for c-Fos induction is. Here we show that a single light pulse induced c-Fos in 47% of transfected pyramidal neurons (Fig. 6D). The fraction of c-Fos expressing cells rose to 57% for 5 stimulation pulses and to 65% for 10 or more pulses. c-Fos induction was blocked by TTX but not by AMPA or NMDA receptor antagonists, suggesting that c-Fos upregulation requires action potential firing, but not excitatory synaptic input. This is consistent with studies showing that Ca^2+^ influx through voltage-gated calcium channels can trigger c-Fos expression [23], [24]. In our cell-attached recordings, 54% of cells fired a spike train in response to the first light stimulation, a success rate that fits well to 47% of cells expressing c-Fos after a single light pulse. Our data thus suggests that a single train of 30 or more APs is sufficient for c-Fos induction in hippocampal pyramidal cells. Average c-Fos expression levels increased with the number of light pulses, but reached a plateau after 10 pulses. Again, this observation can be readily explained by the failure of most cells to produce more than 10 spike trains in succession. Stimulation by 50 mM K^+^ induced c-Fos at levels ∼5-fold higher than after light stimulation (Fig. 6A, D), indicating that the dynamic range of the c-Fos-system is very large and might only become saturated under pathophysiological conditions (i.e. persistent depolarization). In conclusion, we show that ChR2(C128A)-mediated firing induces c-Fos expression in a graded and cell-specific manner. This property could be exploited to drive transgene expression in selected cells with a high degree of temporal control.

## Methods

### Cell Culture and Transfection

Hippocampal slice cultures from rats (Sprague Dawley) or fosGFP transgenic mice (C57BL/6) were prepared at postnatal day 4–5 as described [25], according to the rules of the Federal Veterinary Office of Basel-Stadt. After 6–8 days in culture, we used a Helios gene gun (Bio-Rad) to co-transfect individual cells with DNA encoding ChR2(C128X) and tdimer2 (dimeric RFP), each subcloned into a neuron-specific (synapsin 1) expression vector. The C128X point mutations were introduced into wt ChR2 by site-directed mutagenesis. To achieve high expression levels, 5 mg colloidal gold (1.6 µm, Bio-Rad) was coated with 4 µg of each co-transfected construct.

### Electrophysiology

The recording setup was based on a BX-51 microscope equipped with a LUMPlan 60x 0.9NA water immersion objective (Olympus) and a cooled CCD camera (Sensicam QE). For patch-clamp recordings, we used a MultiClamp 700B amplifier (Axon Instruments) controlled by ScanImage [26] and MP-225 manipulators (Sutter Instrument). Experiments were conducted at 29–31°C 1–4 weeks after transfection under low ambient light conditions. To ensure that all experiments started with a uniform dark-adapted channel population, we recorded only from one cell per culture. Artificial cerebrospinal fluid (ACSF) contained (in mM) 119 NaCl, 26.2 NaHCO_3_, 11 D-glucose, 2.5 KCl, 4 MgCl_2_, 4 CaCl_2_, 1 NaH_2_PO_4_. ACSF was complemented with 1 µM TTX, 10 µM NBQX, 10 µM bicuculline for photocurrent measurements in voltage-clamp; 10 µM NBQX for current-clamp or cell-attached recordings. Pipettes for cell-attached experiments contained 150 mM NaCl. Glass pipettes for patch-clamp recordings were filled with intracellular solutions containing (in mM): 135 potassium gluconate, 10 HEPES, 4 MgCl_2_, 4 Na_2_-ATP, 0.4 Na-GTP, 10 Na_2_-phosphocreatine, and 3 ascorbate.

### Photostimulation

EGFP (Chroma #41017; 470/40 exciter) and rhodamine filter sets (Zeiss #43; 545/25 exciter) were used for arc lamp stimulation. Light pulses (50 ms if not indicated otherwise) were controlled by a mechanical shutter (Uniblitz). Light pulse power (measured in the back focal plane of the objective) was 8.4 mW for blue and 44 mW for green pulses, if not indicated otherwise. In electrophysiological experiments, a minimal interval of 2.5 min was maintained between stimulation pulses. For stimulation in the cell culture incubator, culture inserts in 35 mm cell culture dishes were illuminated from below with blue LEDs (Luxeon V Star blue, Philips; 0.2 mW/mm^2^ at 15 mm from the emitter).

### Immunohistochemistry

For c-Fos immunolabeling cultures were fixed at the indicated intervals after onset of stimulation using Formal-Fixx (Shandon). The primary antibody (Anti-c-Fos rabbit pAb, Calbiochem) was applied over-night in permeabilization buffer (PBS containing 1% BSA, 0.3% Triton X-100). After extensive washing the secondary antibody (Alexa Fluor 488 goat anti-rabbit IgG, Invitrogen) was applied for 2 h in 1∶3 diluted permeabilization buffer. After extensive washing cultures were mounted on glass slides and stored at 4°C.

### Data Analysis

For analysis of c-Fos expression, transfected cells were selected based on the RFP signal. For unbiased analysis of c-Fos expression levels, the nuclear c-Fos signal (green fluorescence) was measured and background subtracted using ImageJ. The experimenter was blind to the experimental conditions. Electrophysiological recordings were analyzed with custom software written in MATLAB. For characterization of spike trains, the initial frequency is the frequency of the first two spikes fired in a burst and the instantaneous frequency is the frequency between two subsequent spikes. Numerical values are given as mean±s.e.m. Statistics were performed using two-tailed t tests (Bonferroni-corrected for multiple comparisons).
